# Barth syndrome cardiomyopathy: targeting the mitochondria with elamipretide

**DOI:** 10.1007/s10741-020-10031-3

**Published:** 2020-10-01

**Authors:** Hani N. Sabbah

**Affiliations:** Department of Medicine, Division of Cardiovascular Medicine, Henry Ford Hospital, Henry Ford Health System, 2799 West Grand Boulevard, Detroit, MI 48202 USA

**Keywords:** Barth syndrome, Mitochondria, Cardiomyopathies, Electron transport chain, Adenosine triphosphate

## Abstract

Barth syndrome (BTHS) is a rare, X-linked recessive, infantile-onset debilitating disorder characterized by early-onset cardiomyopathy, skeletal muscle myopathy, growth delay, and neutropenia, with a worldwide incidence of 1/300,000–400,000 live births. The high mortality rate throughout infancy in BTHS patients is related primarily to progressive cardiomyopathy and a weakened immune system. BTHS is caused by defects in the TAZ gene that encodes tafazzin, a transacylase responsible for the remodeling and maturation of the mitochondrial phospholipid cardiolipin (CL), which is critical to normal mitochondrial structure and function (i.e., ATP generation). A deficiency in tafazzin results in up to a 95% reduction in levels of structurally mature CL. Because the heart is the most metabolically active organ in the body, with the highest mitochondrial content of any tissue, mitochondrial dysfunction plays a key role in the development of heart failure in patients with BTHS. Changes in mitochondrial oxidative phosphorylation reduce the ability of mitochondria to meet the ATP demands of the human heart as well as skeletal muscle, namely ATP synthesis does not match the rate of ATP consumption. The presence of several cardiomyopathic phenotypes have been described in BTHS, including dilated cardiomyopathy, left ventricular noncompaction, either alone or in conjunction with other cardiomyopathic phenotypes, endocardial fibroelastosis, hypertrophic cardiomyopathy, and an apical form of hypertrophic cardiomyopathy, among others, all of which can be directly attributed to the lack of CL synthesis, remodeling, and maturation with subsequent mitochondrial dysfunction. Several mechanisms by which these cardiomyopathic phenotypes exist have been proposed, thereby identifying potential targets for treatment. Dysfunction of the sarcoplasmic reticulum Ca^2+^-ATPase pump and inflammation potentially triggered by circulating mitochondrial components have been identified. Currently, treatment modalities are aimed at addressing symptomatology of HF in BTHS, but do not address the underlying pathology. One novel therapeutic approach includes elamipretide, which crosses the mitochondrial outer membrane to localize to the inner membrane where it associates with cardiolipin to enhance ATP synthesis in several organs, including the heart. Encouraging clinical results of the use of elamipretide in treating patients with BTHS support the potential use of this drug for management of this rare disease.

## Introduction

Barth syndrome (BTHS) is a rare, X-linked recessive disorder characterized by cardiolipin abnormalities, skeletal muscle weakness, abnormal mitochondria, neutropenia, growth retardation, and cardiomyopathy [1]. Current estimates are that the incidence of BTHS is 1/300,000–400,000 live births, with 111 diagnosed individuals in the USA and 230–250 worldwide, though it is widely accepted that the disease is underdiagnosed [2]. Biopsies of the heart, liver, and skeletal muscle of patients with BTHS showed both mitochondrial malformations and dysfunction. BTHS patients have a high mortality rate throughout infancy that is primarily related to progressive cardiomyopathy and a weakened immune system [2].

BTHS was first described in 1983 [3]. Identification of the underlying genetic culprit in 1996 [4–7] led to identification of a large number of affected patients [1]. BTHS is caused by mutations in a recessive, X-linked gene located in the distal region of chromosome Xq28. This gene encodes the tafazzin enzyme, a transacylase involved in cardiolipin side chain remodeling. Tafazzin is critical for the maturation of cardiolipin, an essential phospholipid of the inner mitochondrial membrane. In BTHS, the significant loss of mature cardiolipin leads to profound derangement in mitochondrial structure and function. Accordingly, mitochondria are a key therapeutic target in the treatment of BTHS patients. The first of two segments of the discussion that follows will focus on the pathophysiology of BTHS. This includes mechanistic insights into the disease, a description of the myopathic and inflammatory pathologies associated with BTHS, and an overview of current and potential new therapies. The second segment will focus on elamipretide, a novel mitochondria-targeting peptide that is a promising therapeutic agent for BTHS.

## Mitochondrial bioenergetics

**Fig. 1:**
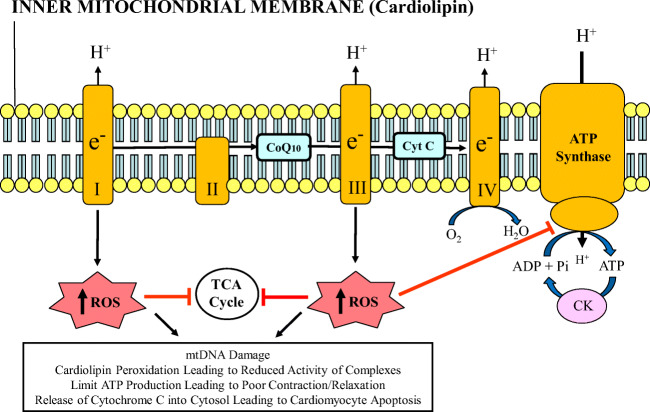
Depiction of mitochondrial inner membrane and electron transport chain consisting of complexes I through V. Reactive oxygen species (ROS) are generated at complexes I and III. Excessive ROS production can lead to mitochondrial and cardiomyocyte dysfunction by inhibiting the tricarboxylic acid (TCA) cycle enzymes and adenosine triphosphate (ATP) synthase, and by damaging mtDNA. Adapted with permission from reference 8. CK, creatine kinase; CoQ10, coenzyme Q10; Cyt C, cytochrome c; mtDNA, mitochondrial DNA; Pi, inorganic phosphate

Mitochondria are an intracellular double-membraned network that are the “power grids” of eukaryotic cells. Mitochondria are most abundant in cells with high energy demands, notably striated muscles. The heart is the most metabolically active organ in the body and possesses the highest content of mitochondria of any tissue [9], comprising about 25% of cell volume in the human myocardium [10, 11] and approximately 35% of cardiomyocyte volume [12]. The key role of mitochondria in muscle cells is the regeneration of adenosine triphosphate (ATP) from adenosine diphosphate (ADP) using macromolecular complexes that form the electron transport chain (ETC). These protein complexes are imbedded in the mitochondrial inner membrane, and include the following: nicotinamide-adenine dinuculeotide (NADH) dehydrogenase (complex I), succinate dehydrogenase (complex II), cytochrome bc_1_ (complex III), and cytochrome c oxidase (complex IV) [12]. As electrons flow energetically “down-hill” through the ETC (ultimately reducing oxygen to water), protons are pumped from the matrix into the cristae lumen. This establishes an electrochemical proton gradient characterized by a highly negative mitochondrial membrane potential. The re-entry of protons into the matrix through the ATP synthase (complex V) provides the energy to regenerate ATP from inorganic phosphate (Pi) and ADP (Fig. 1) [13–15]. The coupling of substrate oxidation and ATP formation in the mitochondria, termed oxidative phosphorylation, is central to tissue and organ health [12]. Cardiolipin is a unique phospholipid expressed almost exclusively in the inner mitochondrial membrane and involved in nearly every aspect of mitochondrial structure and function.

Humans produce and consume about 65 kg of ATP every day, with the heart accounting for about 8% of ATP consumption daily, or about 6 kg [16]. About 90% of cellular ATP within the myocardium is used to meet the enormous energy requirements for contraction and relaxation, both of which are ATP-dependent [17]. Mitochondrial dysfunction therefore plays a central role in a wide variety of metabolic and cardiac disorders, including heart failure (HF) and BTHS [1, 18], the subject of this review. Dysfunctional mitochondria in skeletal muscle has been implicated in HF-associated exercise intolerance [19] and in skeletal muscle myopathy and exercise intolerance in BTHS [20].

Because ATP cannot be stored, it is critical that the rate of ATP synthesis matches the rate of ATP consumption [8]. This process is accomplished by mitochondrial oxidative phosphorylation within the ETC using fatty acids as the primary fuel source [21]. Although there are numerous reasons why human hearts fail, a mismatch between ATP supply and demand has been observed in almost all etiologies of HF [16]. Changes in oxidative phosphorylation are characterized by decreased energy production, with reductions in oxygen utilization, and respiratory chain and ATP synthase activity. Mitochondrial dysfunction also contributes to skeletal muscle performance limitations by reducing the ability of mitochondria to meet the ATP demands of aerobic, slow-twitch, fatigue-resistant working muscles [22]. Lack of availability of energy during activity leads, in part, to exercise intolerance, a characteristic feature of both acquired forms of HF and BTHS [23].

## Tafazzin, TAZ gene mutations, and Barth syndrome

The TAZ gene provides instructions for producing the enzyme tafazzin. Since tafazzin transacylase activity is responsible for cardiolipin remodeling, it is critical to maintaining mitochondrial inner membrane structure and function. Tafazzin is encoded by the TAZ gene, is highly expressed in cardiac and skeletal muscle, and functions as a phospholipid-lysophospholipidtransacylase in humans [6, 7]. The protein is produced by alternate splicing of the TAZ gene or *G4.5*. The gene is a single-copy gene composed of ∼ 11 kb of genomic DNA with 11 exons, which maps to Xq28, and two ATG initiation sites. Multiple mRNAs can be produced by alternate splicing at exons 5–7, resulting in tafazzin proteins ranging from 129 to 292 amino acids in length that differ at the N-terminal and central regions. Two putative functional domains have been identified: (1) a highly hydrophobic segment of 30 residues at the N-terminus, which acts as a membrane anchor, and (2) a hydrophilic segment in the central region that forms an exposed loop interacting with other proteins.

As alluded to earlier, tafazzin is a nuclear-encoded acyltransferase that is “trafficked” to the inner mitochondrial membrane and functions in remodeling cardiolipin fatty acyl chains [24]. The putative phospholipid-binding site, which is the active site of tafazzin, is a 57-amino acid cleft with two open ends and positively charged residues [25]. Tafazzin has at least 4 different isoforms and has a molecular weight of approximately 35 kDa, but may also appear in lower molecular weights due to species differences in isoform expression [4, 5]. The TAZ gene contains two peptides independent of its active site for directing the protein to the mitochondria, forming residues 84–95 in exon 3 and residues 185–200 in exon 7/8 [26]. Within the mitochondria, tafazzin localizes between the inner mitochondrial membrane and outer mitochondrial membrane, facing the intermembrane space [27, 28]. Tafazzin’s characteristic interfacial anchoring is achieved by its hydrophobic sequence from residues 215–232 [29]. Finally, the translocase of the outer membrane and the translocase of the inner membrane mediate tafazzin’s movement and insertion into the outer mitochondrial membrane and anchoring to the inner mitochondrial membrane [29].

Mutations in the TAZ gene have been associated with mitochondrial dysfunction in BTHS cardiomyopathies, dilated cardiomyopathy, hypertrophic-dilated cardiomyopathy, endocardial fibroelastosis, LV noncompaction, breast cancer, papillary thyroid carcinoma, non-small cell lung cancer, glioma, gastric cancer, thyroid neoplasms, and rectal cancer [30–32]. Several functional classes of TAZ gene mutations have been classified based on the pathogenic loss-of-function mechanisms of each mutation. A variety of *TAZ* gene mutations, including splice site mutations, insertions, deletions, nonsense, and missense mutations, located throughout the *TAZ* gene have been reported to cause BTHS. Individuals harboring mutations in the TAZ gene, namely patients with BTHS, manifest compromised or missing tafazzin enzymatic activity, resulting in specific alterations in cardiolipin that include increased molecular species heterogeneity, decreased levels of cardiolipin, and increased levels of monolysocardiolipin. If, for example, a given TAZ gene mutation gives rise to a tafazzin protein with residual enzyme activity, lesser changes in cardiolipin content and composition, and a milder phenotype, may be anticipated [33]. On the other hand, more severe mutations in the TAZ gene that lead to complete loss of transacylase activity will have a more deleterious effect on inner mitochondrial membrane structure and function. Despite this, TAZ gene mutations have been described in which the correlation between genotype and phenotype is not apparent [34]. Conceivably, individual differences in TAZ mRNA splicing and mRNA stability or differences in tafazzin protein turnover rate could affect residual enzyme activity. Similarly, one or more extraneous phenotypic modifiers, ranging from environmental to biochemical, could be responsible for differences in the degree of disease manifestation [33, 34]. Despite the X-linked inheritance of Barth’s syndrome and the identification of many females carrying *TAZ* gene mutations, there are no reports of females with the classic pathology observed in BTHS.

## Cardiolipin and Barth syndrome

Among mitochondrial lipids, cardiolipin is unique. It is the only phospholipid that is specific to mitochondria. Cardiolipin accounts for roughly 20% of inner membrane phospholipids, and plays a vital role in the molecular organization and physiological function of mitochondria. Cardiolipin is very abundant in mitochondria of myocytes of the heart and skeletal muscle. Unlike other glycerophospholipids, cardiolipin is unique in that two phosphatidate moieties share the same glycerol head group, giving rise to an anionic phospholipid with four esterified fatty acyl chains and a cone-shaped structure [35]. In myocytes and all other eukaryotes, cardiolipin is confined to the inner mitochondrial membrane. In cardiomyocytes and skeletal muscle, nearly 90% of cardiolipin exists as a single molecular species, tetralinoleoylcardiolipin [36]. Formation of tetralinoleoylcardiolipin depends largely on a series of phospholipid remodeling reactions, with tafazzin being one of the last transacylases leading to mature cardiolipin [37]. In addition to tafazzin, cardiolipin remodeling can occur via the endoplasmic reticulum localized enzyme acyl-CoA:lysocardiolipin acyltransferase (ALCAT1) [38, 39] and via mitochondrial monolysocardiolipin acyltransferase (MLCAT) [40, 41]. Even though the relative contribution of ALCAT1 and MLCAT to the total pool of mature cardiolipin is not known, it is well recognized that ALCAT1- and MLCAT-mediated reactions cannot fully compensate for loss of tafazzin [35]. Tafazzin catalyzes remodeling of immature cardiolipin to its mature composition containing a predominance of tetralinoleoyl moieties. The condensation of phosphatidylglycerol and CDP-diacylglycerol produces immature cardiolipin which contains predominantly shorter chain saturated and monounsaturated fatty acyl chains (palmitic and oleic acids). The immature cardiolipin is then remodeled to form mature cardiolipin through the coordinated activity of specific lipases and acyltransferase/transacylase, primarily tafazzin [42–44]. Mature cardiolipin is responsible for maintenance of mitochondrial membrane fluidity, osmotic stability, and proper curvature of the cristae, which serves as a crucial binding site for electron transport chain (ETC) proteins and stabilization of the ETC supercomplexes [6, 45–47].

Cardiolipin plays essential roles in mitochondrial protein/metabolite transport, mitochondrial morphology, and mitochondrial bioenergetics [1]. Nuclear-encoded mitochondrial proteins are transported into mitochondria after translation in the cytosol. Protein transport across mitochondrial membranes is mediated by specialized translocases that are located in cardiolipin-rich contact sites where the inner and outer membranes converge to close proximity [1, 48, 49]. Mitochondrial morphology is markedly abnormal when cardiolipin is deficient, as in BTHS, where marked malformation of cristae structures is evident [1, 50]. Similar abnormalities of mitochondrial morphology have been reported in many tafazzin-deficient cell and animal models [1].

Cardiolipin regulates several enzyme activities and, in particular, those related to oxidative phosphorylation and coupled respiration [51–56]. Cardiolipin binds complexes I, III, IV, and V and stabilizes the super complexes (I/III/IV and II/III/IV), indicating an absolute requirement of cardiolipin for catalytic activity of these complexes [52, 53, 57, 58]. The activity of numerous respiratory chain complexes is directly related to cardiolipin content [54, 59, 60] and composition [55, 56, 61]. Under normal physiological conditions, monolysocardiolipin is converted to mature cardiolipin by a functional tafazzin enzyme. Impaired tafazzin activity in BTHS leads to the accumulation of monolysocardiolipin and an overall loss in cardiolipin species. Both of these contribute to morphological abnormalities of the mitochondrial inner membrane and inefficient cellular bioenergetics [62–66].

## Cardiomyopathies of Barth syndrome

Cardiac disease is common in Barth syndrome and is often diagnosed within the first year of life. In fact, cardiomyopathy is the single most frequent sign, occurring in approximately 90% of males with BTHS, although the manifestation and severity vary for each individual. Several cardiomyopathic phenotypes have been described. Dilated cardiomyopathy is common and is characterized by decreased left ventricular (LV) systolic function, increased LV mass, and an increased LV end-diastolic dimension [23, 67, 68]. Left ventricular noncompaction is also commonly seen either alone or in conjunction with other cardiomyopathic phenotypes and is characterized by LV trabeculations with associated wall motion abnormalities [68]. Endocardial fibroelastosis may be seen, although less commonly [69]. Hypertrophic cardiomyopathy [70], as well as an apical form of hypertrophyic cardiomyopathy [71], is also reported to occur in BTHS. A mixed hypertrophic-dilated cardiac phenotype characterized by thickening of the LV walls with an increase in LV mass and end-diastolic dimension, and depressed systolic function has also been reported [72]. Transition between distinct phenotypes has also been described in the setting of LV noncompaction termed “undulating phenotype” [73]. No current mechanism has been proposed that explains the various cardiomyopathic phenotypes seen in BTHS, yet evidence of varying phenotypic cardiac disease is well documented in families with recognized sarcomeric mutations suggesting shared molecular etiology of different forms of cardiomyopathy [74]. In addition, there is an increased risk of cardiac arrhythmia in BTHS, some of which may be life-threatening. The arrhythmia may be a direct result of abnormal mitochondrial function and/or a function of the associated cardiac phenotype, as ventricular arrhythmias are well reported in dilated cardiomyopathy, LV noncompaction, and hypertrophic cardiomyopathy [67, 75, 76].

## Ca^2+^-ATPase, LV diastolic function, and Barth syndrome

The sarcoplasmic reticulum Ca^2+^-ATPase (SERCA) is a P-type ATPase which catalyzes the active transport of Ca^2+^ ions from the cytoplasm into the sarcoplasmic reticulum. In cardiac muscle, the primary isoform is SERCA2a, which is necessary for the proper regulation of muscle contraction and, importantly, muscle relaxation, by ensuring proper Ca^2+^ uptake into the sarcoplasmic reticulum during diastole and presence of sufficient Ca^2+^ load in the sarcoplasmic reticulum for systolic contraction. Phospholamban is a 52-amino acid protein that, in its non-phosphorylated state, binds to and regulates SERCA by decreasing its affinity for Ca^2+^ [77, 78]. When phosphorylated at serine 16 and threonine 17 by protein kinase A and Ca^2+^-calmodulin-dependent protein kinase II, respectively, phospholamban dissociates from SERCA, thereby restoring its affinity for Ca^2+^ [78].

In addition to phospholamaban, SERCA can be regulated by reactive oxygen/nitrogen species (ROS/RNS) [79–81]. The SERCA pump is highly susceptible to oxidative and nitrosative post translational modification as they contain vulnerable cysteine, lysine, and tyrosine residues [79–81]. Under conditions of oxidative stress, superoxide and nitric oxide react to form peroxynitrite, which can then adduct to tyrosine resulting in altered SERCA protein structure and function [82]. Interestingly, SERCA dysfunction, SERCA2a tyrosine nitration, increased ROS formation, and phospholamban dysregulation have all been implicated in cardiomyopathy, a key manifestation of Barth syndrome [67, 83–85]. It was previously shown that cardiolipin is markedly decreased in tafazzin knockdown mice, a model of Barth syndrome, leading to mitochondrial dysfunction and elevated ROS and RNS levels [86, 87]. In tafazzin knockdown mice, SERCA activity was impaired and SERCA2a tyrosine nitration increased compared with wild-type mice [88]. SERCA2a tyrosine nitration was negatively correlated with maximal SERCA activity [88]. These abnormalities were likely due to mitochondrial dysfunction and increased oxidative stress [88] and can promote LV diastolic dysfunction and, subsequently, systolic dysfunction. Left ventricular diastolic dysfunction has been shown in mice models of tafazzin knockdown that manifest noncompaction even in the absence of myocardial fibrosis and myocardial hypertrophy and speculated to result from alterations in cardiolipin and abnormal Ca^2+^ homeostasis [87]. Patients with myocardial noncompaction, typically present with BTHS, almost always exhibit diastolic dysfunction [89]. Left ventricular diastolic dysfunction with preserved systolic function has been demonstrated in the Friend of GATA-2 (FOG-2) null mouse that also develops noncompaction [90]. These findings suggest that SERCA may be a viable therapeutic target for BTHS.

## Inflammatory cytokines and Barth syndrome

Inflammatory cytokines are signaling molecules produced predominantly by T-helper cells and macrophages and are involved in the upregulation of inflammatory reactions [91]. By initiating the inflammatory response, cytokines regulate the host defense against pathogens mediating the innate immune response. Several reports have demonstrated enhanced expression and release of inflammatory cytokines such as tumor necrosis factor alpha (TNF-α) and interleukin-6 (IL-6) in patients with acquired HF [92–94]. Cytokines and other inflammatory mediators may contribute to the development and progression of systolic HF. This pathogenic role of inflammatory cytokines in chronic HF is supported by various studies in animal models [95–98]. Systemic administration of TNF-α in concentrations comparable to those found in the circulation of HF patients has been shown to induce a dilated cardiomyopathy-like phenotype in animal models [95], and cardiac-specific overexpression of TNF-α has been found to promote a phenotype mimicking several features of clinical HF such as cardiac hypertrophy, ventricular dilation and fibrosis, and several biochemical and cellular dysfunctions [96]. More recent studies in gene-modified mice have also shown a link between IL-6 and the development of HF [97]. Inflammatory cytokines may modulate myocardial function by a variety of mechanisms including stimulation of hypertrophy and fibrosis through direct effects on cardiomyocytes and fibroblasts, and impairment of myocardial contractile function through direct effects on intracellular calcium transport. Furthermore, inflammation-mediated signal transduction through adrenergic receptors, induction of apoptosis, and stimulation of genes involved in myocardial remodeling [98] can exacerbate pathology. While increased inflammation is a well-known feature of systolic heart failure, this has been less well studied in patients with HF and preserved ejection fraction (HFpEF). Recent studies, however, have shown that patients with overt HFpEF frequently have increased plasma levels of TNF-α and IL-6 [99]. Moreover, IL-6 infusion in rats results in concentric LV hypertrophy, increased collagen volume fraction, and increased myocardial stiffness [100], all characteristic features of HFpEF.

Given that BTHS is characterized by cardiomyopathies, it would not be surprising to expect increased levels of TNF-α and IL-6 in this disease state. Elevated levels of TNF-α and IL-6 have indeed been reported in BTHS patients, further exacerbating their cardiomyopathy [101]. In addition to the adverse effects of cytokines on the myocardium as eluded to earlier, increased expression of cytokines can have an adverse effect on growth anomalies widely reported in BTHS. There has been growing evidence that inflammatory processes may influence normal muscle development in children [100]. Increased levels of TNF-α have been shown to suppress the AKT/mTOR (mammalian target of rapamycin) pathway, a crucial pathway for regulating skeletal muscle hypertrophy, thereby increasing muscle catabolism [102–104]. Inflammatory cytokines may also antagonize the anabolic effects of insulin-like growth factor (IGF), a known promoter of muscle hypertrophy [105–107]. It is plausible that an active inflammatory process can contribute to the growth abnormalities and pathology observed in BTHS. Higher levels of IL-6 and lower IGF-1 levels were observed in Barth syndrome patients compared with age-matched controls [101]. This finding may implicate inflammatory processes in the catabolic nature of BTHS pathology, as well as provide a link to mitochondrial dysfunction. Furthermore, lower levels of IGF-1 may contribute to some of the growth delays and myopathies observed in Barth syndrome [101].

### Cytokines and mitochondrial damage-associated molecular patterns

Functional, ultrastructural, and dynamic abnormalities of mitochondria occur in BTHS and can lead to cellular stress and death. In recent years, much has been unraveled about the pro-inflammatory properties of various mitochondrial molecules once released from the mitochondrial compartment and into the cytosol or the extracellular space [108, 109]. Mitochondria can generate and release multiple molecules that can stimulate the innate immune system. On entering the cytoplasm or the extracellular space, mitochondrial damage-associated molecular patterns (DAMPs; also known as mitochondrial alarmins) can become pro-inflammatory and initiate innate and adaptive immune responses by activating cell surface and intracellular receptors [108, 109]. Among the molecules listed as mitochondrial DAMPs are *N*-formyl peptides, cardiolipin, and mitochondrial DNA (mtDNA), which are released from damaged mitochondria and can activate sterile inflammation [109–112]. Inflammatory responses induced by sterile stimuli can elicit recruitment of neutrophils and macrophages, production of inflammatory cytokines and chemokines, and induction of T cell-mediated adaptive immune responses [113]. Of note, mtDNA has recently been established as an important DAMP and a possible trigger of various inflammatory or degenerative diseases [114, 115]. Failure to remove the damaged mitochondria with resulting leak of DAMPs has been proposed as an underlying mechanism in the pathophysiology of HF [8].

## Skeletal muscle and Barth syndrome

Exercise intolerance is a hallmark of most, if not all, cardiomyopathies, including Barth syndrome, making it nearly impossible for individuals with BTHS to perform activities of daily living or pursue an acceptable quality of life [116]. In patients with chronic acquired HF, exercise intolerance has been attributed to skeletal muscle atrophy, a shift from slow-twitch, fatigue-resistant, type 1 (oxidative) to fast-twitch type 2 (glycolytic) muscle fibers and to mitochondrial abnormalities [117]. A decrease in the relative composition of type 1 fibers and an increase in type 2 fibers have been shown in an animal model of HF and were associated with reduced exercise tolerance [118]. Changes in composition of skeletal muscle fiber type have also been described in patients with HF and were also associated with exercise intolerance [119–121]. These patients also manifest a reduction in myosin heavy-chain type I [121], an isoform that is more abundant in skeletal muscle type 1 aerobic fibers. The shift in fiber-type composition may be partly due to skeletal muscle mitochondrial abnormalities and the associated reduction of ATP synthesis needed by aerobic type 1 fibers [122]. A reduction in ATP production can lead to an adaptation of slow-twitch type 1 fibers to utilizing glycogen as their energy source and thus shifting fiber-type composition toward a fast-twitch phenotype [117]. Under normal physiologic conditions, ATP production by oxidative phosphorylation in the mitochondria fulfills most of the ATP demands of skeletal muscle at rest and during exercise [22]. Mitochondrial dysfunction, therefore, can contribute to decrements in skeletal muscle performance via loss of mitochondrial capacity to generate ATP, or reduced ability to meet the ATP demands of working skeletal muscle. Abnormalities of skeletal muscle mitochondria have been shown in the skeletal muscle of dogs with experimental HF compared with normal dogs [19] as evidenced by reduction in ADP-stimulated respiration, membrane potential, and complex IV [115]. A variety of alterations specific to skeletal muscle, including muscle atrophy, fiber-type changes, defects in oxidative metabolism, and decreased mitochondrial volume density, have been described in patients with HF [123]. Studies using ^31^P nuclear magnetic resonance (NMR) spectroscopy clearly demonstrated intrinsic skeletal muscle metabolic abnormalities in patients with chronic acquired HF [124].

Daily functional activities, notably ambulation, require a combination of muscular strength, endurance, and balance. Patients with BTHS present with exercise intolerance that is thought to be due to both cardiac impairment and decreased skeletal muscle oxygen utilization [23]. In patients with BTHS, affected individuals showed signs of multiple skeletal muscle impairments including impaired functional exercise capacity, knee and hip flexor, grip, and extensor strength, and reduced daily activity [125]. In patients with Barth syndrome, ^31^P nuclear magnetic resonance (NMR) spectroscopy showed a higher resting skeletal muscle phosphocreatine to inorganic phosphate ratio (PCr/Pi) compared with control subjects, a finding consistent with skeletal muscle containing large fractions of type 2, fast-twitch, glycolytic fibers and a smaller fraction of type 1, slow-twitch, oxidative fibers [126, 127]. This finding suggests that individuals with BTHS rely on glycolytic metabolism to a greater extent than those without BTHS. Barth syndrome patients also have higher respiratory exchange ratios during exercise [23] and a greater glucose rate of disposal during a hyperinsulinemic-euglycemic clamp procedure [128]; both suggestive of higher glycolytic dependence. This higher glycolytic capacity is likely compensatory for an impaired mitochondrial capacity to generate ATP. Skeletal muscle and cardiac bioenergetics, as determined from post-exercise PCr recovery kinetics using ^31^P-MRS, are markedly impaired in BTHS patients when compared with unaffected, age-matched, sedentary controls and are associated with exercise intolerance [20]. Mitochondrial function in tissues such as myocardium and skeletal muscle is closely integrated with physiological demands below the anaerobic threshold [129, 130]. Mitochondrial respiration has been shown to be impaired in BTHS patients [66] possibly due to supercomplex destabilization [131], higher degradation and reduced levels of mitochondrial cardiolipin [132], abnormal mitochondrial morphology [50, 133], excessive production of reactive oxygen species, and/or defects in ATP synthase activity [66].

## Current therapies for Barth syndrome

Barth syndrome is often considered a lethal early childhood disease. However, improvements in the management of associated neutropenia/infectious risks, skeletal myopathy, and cardiac disease have resulted in improved survival. This is in large part secondary to the treatment of low circulating neutrophil counts and avoidance of infection, systolic dysfunction, and cardiac arrhythmia [71]. Neutropenia is treated with granulocyte colony-stimulating factor (G-CSF) with reasonable success concomitant with appropriate prophylactic antibiotics if clinically indicated. Growth delay is managed with growth hormone (GH) supplementation when central GH deficiency is documented [71]. Arginine depletion has been implicated as contributing to low growth rates in BTHS patients, resulting in an increased use of arginine supplementation as a putative treatment to improve growth rates [134, 135].

Clinically significant arrhythmias can be a major cause of mortality in Barth syndrome. The risk of ventricular arrhythmias is well known and may be precipitated by associated metabolic acidosis or concomitant LV systolic dysfunction [136]. Ventricular arrhythmias in the form of ventricular tachycardia or ventricular fibrillation may result in sudden cardiac death. The use of implantable cardioverter defibrillators has been documented in BTHS, although limited data exist regarding the effectiveness of this therapy [137]. Treatment of associated myocardial dysfunction, however, remains paramount to alleviating symptoms as well as prolonging life in patients with BTHS [71]. Medical and surgical options for dilated cardiomyopathy have increased, but remain sparse for other cardiac phenotypes of Barth syndrome. Angiotensin-converting enzyme inhibitors or angiotensin receptor blockers, potentially in combination with approved beta-adrenergic receptor blockers [71], are commonly used to treat BTHS cardiomyopathy. For those patients with worsening HF, more aggressive therapies may be needed in the form of intravenous agents such as vasodilators or inotropes, left ventricular assist devices, and/or cardiac transplantation [138–140].

In a single-center experience, 4 BTHS patients (average age ~ 2 years) underwent orthotopic heart transplantation [141]. At the time of the follow-up report, patients were alive with average age of ~ 8.6 years. The patients did not demonstrate an increased rate of rejection compared with the general heart transplant population [141].

## Novel experimental therapeutic approaches for treating Barth syndrome

Developing effective therapies for Barth syndrome continues to be a challenge, especially because of the limited number of patients, extraordinary phenotypic variability, and unpredictable clinical course. Experimental and potentially useful therapies include recombinant adenovirus-associated TAZ gene overexpression, peroxisome proliferator-activated receptor (PPAR) agonist, and antioxidants. Recombinant adeno-associated virus (rAAV) vectors are utilized in gene therapy approaches because gene delivery is essentially a nonpathogenic virus that elicits a minimal immune response and persists for long periods of time as an episome within the nucleus of cells, providing stable gene transfer without disruption of genes, by insertional mutagenesis [142]. In a recent study aimed at developing a clinically relevant gene therapy to restore tafazzin function and treat Barth syndrome, AAV-mediated TAZ gene replacement was shown to restore mitochondrial and cardioskeletal function in a Barth syndrome mouse model of TAZ gene knockdown [24]. Due to a central role in energy metabolism and mitochondrial bioenergetics, peroxisome proliferator-activated receptors (PPARs) have been considered potential therapeutic targets to ameliorate cardiac dysfunction induced by tafazzin deficiency. Beneficial effects of activation of the PPAR/PGC1α axis have been demonstrated in various mitochondrial disorders [143]. The PPAR pan-agonist bezafibrate has been shown to ameliorate cardiomyopathy in a mouse model of Barth syndrome [143]. Dysfunction of mitochondria increases ROS production and contributes to cardiac dysfunction in both acquired HF and BTHS [144]. The mitochondria-targeted antioxidant, mito-Tempo, was shown to prevent cardiac dysfunction induced by tafazzin gene knockdown in cardiac myocytes [144]. The above discussed therapies, while promising, must await confirmation of potential benefit by clinical trials in patients with BTHS.

## Elamipretide: potential novel therapy for Barth syndrome

Elamipretide (also known as SS-31, MTP-131, Bendavia™) is a water-soluble, aromatic-cationic mitochondria-targeting tetrapeptide that readily penetrates and transiently localizes to the inner mitochondrial membrane (7115). Elamipretide crosses the mitochondrial outer membrane and localizes to the inner membrane where it associates with cardiolipin, improving membrane stability and ATP production and reducing pathogenic ROS production. Cardiolipin plays a central role in cristae formation, mitochondrial fusion, mtDNA stability and segregation, and function and organization of the respiratory complexes into supercomplexes for oxidative phosphorylation. Elamipretide has been shown to enhance ATP synthesis in multiple organs, including the heart, kidney, neurons, and skeletal muscle [145–149]. High-resolution respirometry of individual electron transport chain complexes in permeabilized ventricular fibers from ischemia-reperfusion rats showed that ischemia-reperfusion-induced decrements in mitochondrial complexes I, II, and IV were significantly alleviated with elamipretide [150]. Furthermore, studies in serial block face scanning electron microscopy used to create high-resolution 3-dimentional reconstructions of cristae ultrastructure showed that disease-induced fragmentation of cristae networks was improved with elamipretide [150]. Studies using biomimetic membranes modeling the inner mitochondrial membranes also showed that elamipretide improved membrane biophysical properties by aggregating cardiolipin. These studies suggest that mitochondrial structure-function are interdependent and demonstrate that elamipretide targets mitochondrial membranes to sustain cristae networks and improve bioenergetic function [150]. While there are limited data sets with elamipretide in non-clinical BTHS models, it has been studied extensively in models of experimental LV dysfunction and failure. It is these studies in animal models of acquired HF that provide much of the knowledge base available to date that supports the potential use of elamipretide for the treatment of patients with BTHS.

### Modulation of cardiolipin by elamipretide

**Fig. 2:**
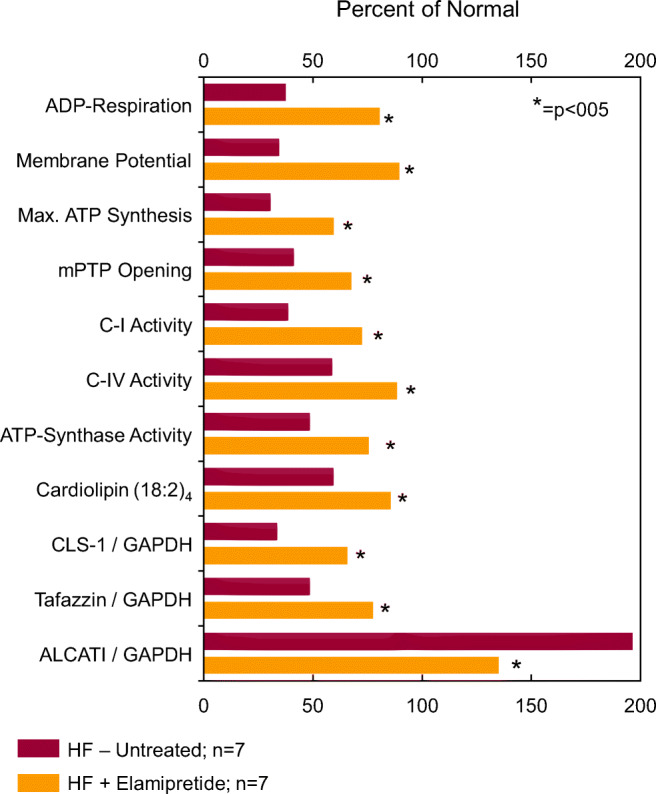
Bar graph depicting magnitude of change of various measures of mitochondrial function calculated as a percent of levels seen in normal dogs (Percent of Normal). The percentages are shown for untreated dogs with coronary microembolization-induced heart failure (HF-Untreated; *n* = 7) and for dogs with heart failure treated with elamipretide (HF+Elamipretide; *n* = 7). Original data in references 163 and 167. The measures are as follows: ADP-dependent state 3 respiration (ADP-Respiration); mitochondrial membrane potential; mitochondrial maximum rate of ATP synthesis (Max. ATP Synthesis); mitochondrial permeability transition pore opening (mPTP Opening); mitochondrial complex I (C-I) activity; mitochondrial complex IV (C-IV) activity; ATP synthase activity; cardiolipin (18:2)_4_; cardiolipin synthase-1 levels (CLS-1) normalized to glyceraldehyde 3-phosphate dehydrogenase (GAPDH) levels; tafazzin levels normalized to GAPDH; and acyl CoA lysocardiolipin acyltransferase-1 (ALCAT1) levels normalized to GAPDH

As noted above, cardiolipin is biosynthesized in a series of steps from phosphatidic acid and remodeled into various species, with muscles containing mostly tetralinolyl cardiolipin (four 18:2 fatty acid chains (18:2)_4_CL). Cardiolipin peroxidation and depletion have been reported in a variety of pathological conditions, including BTHS, and are associated with energy deficiency [151, 152]. In dogs with coronary microembolization-induced HF, total cardiolipin and (18:2)_4_CL were decreased in LV myocardium [152] (Fig. 2), although the decrease in cardiolipin content is more modest (20 to 30%) than that which appears in humans. A 3-month treatment with subcutaneous elamipretide normalized total cardiolipin and (18:2)_4_CL [152]. In these dogs, the decrease in cardiolipin was driven by changes in the lipid structure on the inner mitochondria membrane because of peroxidation and was not necessarily a reflection of changes in the total LV myocardial pool of mitochondrial protein [152]. This observation is also supported by results showing concordant changes in LV myocardial levels of 4-hydroxynonenal, a major bi-product of lipid peroxidation [152].

The effects of elamipretide on cardiolipin were also investigated in freshly explanted failing and nonfailing ventricular tissue from children and adults [153]. Cardiolipin was quantified using liquid chromatography coupled to electrospray ionization mass spectrometry and cardiolipin species were quantified per milligram of protein [153]. The predominant cardiac cardiolipin species, namely (18:2)_4_CL, was reported as a percentage of the total (11 major species were used for total) cardiolipin content. The percentage of (18:2)_4_CL was significantly lower in HF tissue, but acute exposure to elamipretide for 4 hours had no effect on tetralinoleoyl cardiolipin in nonfailing or HF samples [153]. It is likely that the absence of change in cardiolipin after exposure to elamipretide in this study with human tissue was due to the short duration of treatment (4 hours) in comparison with long-term treatment (3 months) with elamipretide in animal models.

Several studies have shown that cardiolipin is decreased in diseases associated with mitochondrial dysfunction and that cardiolipin remodeling enzymes are either upregulated or downregulated [154, 155]. Abnormal protein levels and messenger RNA (mRNA) expression of cardiolipin synthase-1 (CLS-1), an essential enzyme for the synthesis of cardiolipin, and the CL remodeling enzymes tafazzin-1 and acyl-CoA:lysocardiolipin acyltransferase-1 (ALCAT-1) occur in LV myocardium of the failing heart and in cardiomyopathic hearts of patients with BTHS. In LV myocardium of dogs with experimental HF, protein and mRNA levels of CLS-1 were significantly reduced in untreated HF dogs compared with normal dogs [156] (Fig. 2). Protein and mRNA levels of tafazzin-1 were significantly reduced and ALCAT-1 levels significantly increased in untreated HF dogs compared with normal dogs. These changes are directionally similar to those seen in cardiomyopathic hearts of BTHS. Treatment of dogs with HF with subcutaneous injections of elamipretide for 3 months normalized protein and mRNA levels of CLS-1, tafazzin-1, and ALCAT-1 [156] (Fig. 2). Upregulation of CLS-1 and tafazzin-1 and downregulation of ALCAT-1 by elamipretide favor improvement in cardiolipin synthesis and remodeling that can potentially elicit beneficial effects in cardiomyopathic hearts associated with Barth syndrome.

### Effects of elamipretide on mitochondrial dynamics

Mitochondria are a highly dynamic network that constantly undergo biogenesis, fission, fusion, and mitophagy [154, 157]. Fission and fusion are essential for normal mitochondrial function. A number of proteins and lipids have been shown to be important mediators of these dynamic processes [157], particularly peroxisome proliferator-activated receptor gamma coactivator 1α (PGC-1α), which is a transcription factor that drives mitochondrial biogenesis. The failing heart, regardless of etiology, manifests dysregulation in both fission- and fusion-regulating proteins. Downregulation of mitochondrial fusion proteins enhances apoptosis, an important contributor to ongoing cardiomyocyte loss and potential mediator of progressive worsening of the HF state [158–160]. Fission-1 (Fis-1) and dynamin-related protein-1 (Drp-1) are key proteins that regulate mitochondrial fission while mitofusion-2 (Mfn-2) and dominant optic atrophy-1 (OPA-1) are key proteins that regulate mitochondrial fusion [156]. Another key protein in mitochondrial dynamics is mitofilin, a transmembrane protein of the inner mitochondrial membrane that has a critical role in mitochondrial morphology, fission and fusion, and the formation of tubular cristae and cristae junctions [156]. Downregulation of mitofilin can lead to a disorganized mitochondrial inner membrane and ultrastructural abnormalities that are also manifested in the failing heart as well as in cardiomyopathic hearts of patients with Barth syndrome [156]. Studies in LV myocardium of explanted failed human hearts and hearts from dogs with experimental HF showed marked downregulation of the fusion proteins Mfn-2 and OPA-1, and marked upregulation of the fission proteins Fis-1 and Drp-1 [156]. These abnormalities were also accompanied by significant downregulation of PGC-1α and mitofilin [156]. In dogs with experimental HF, long-term therapy with elamipretide normalized PGC-1α, levels of fission and fusion proteins, and protein levels of mitofilin [156]. Taken together, these findings provide support for elamipretide therapy as a positive modulator of mitochondrial structure and dynamics in the setting of HF of various etiologies.

Cardiolipin, through its influence on mitochondrial fission and fusion, also affects mitophagy, a central step in maintaining mitochondrial quality control process and overall health of the cellular mitochondrial pool by removing mitochondria with damage too severe for correction through biogenic or fusion-mediated repair [161]. Suppression of fission, for instance, accelerates mitophagy by lowering the threshold for mitochondrial removal, a maladaptation likely to promote the elimination of functioning mitochondria, while inhibition of fusion suppresses mitophagy, thus reducing the removal of toxic, ROS-producing mitochondria. Studies in mitochondrial fission- and fusion-defective murine hearts and cells showed that Drp-1-mediated mitochondrial fission is essential to properly target mitophagy and restrain mitochondrial permeability transition pore (MPTP)-mediated cell necrosis [162]. Mfn-2 deletion, on the other hand, resulted in accumulation of defective mitochondria without appropriately increasing mitophagy, while Drp-1 ablation interrupted mitochondrial fission by increased mitophagy, causing a generalized loss of mitochondria [162]. MPTP opening in Drp-1-null mitochondria was associated with mitophagy, cardiomyocyte necrosis, and dilated cardiomyopathy [161]. Normalization of cardiolipin synthesis and remodeling in the failing heart along with normalization of fission and fusion protein levels through treatment with elamipretide argues well for maintenance of essential physiologic levels of mitophagy.

### Effects of elamipretide on mitochondrial function

Functional abnormalities of mitochondria exist in most if not all forms of HF and cardiomyopathies including Barth syndrome. In the failing heart, mitochondrial functional abnormalities are characterized by poor respiration, opening of the permeability transition pores (mPTP), collapse of mitochondrial membrane potential, reduced rate of ATP synthesis, and excessive production of ROS [152, 156, 163–165]. These abnormalities are typically associated with abnormal synthesis and remodeling of cardiolipin and with increases in cytosolic cytochrome c [152, 166, 167]. The combination of ROS with elevated mitochondrial calcium concentrations leads to mPTP opening and subsequently reduced membrane potential, which decreases the extra-mitochondrial phosphorylation potential, adversely impacting cell function [168, 169]. A surge in ROS production can lead to cytochrome c release, which initiates cell death via apoptosis through activation of caspase-3 [152, 170]. ROS can damage the mitochondrial electron transport chain [171–174], trigger lipid peroxidation [175], and cause DNA strand breaks [176], all of which can lead to mitochondrial dysfunction [177]. Elevated mitochondrial ROS production and downstream ROS-mediated damage, mtDNA damage, and defects in electron transport complexes have been reported in animal models of HF [178–180]. Mitochondrial damage to cardiomyocytes from excess ROS production limits ATP production to a level insufficient to support contractile function during times of high oxidative energy requirements [172]. Mitochondria of the failing heart also manifest significant reductions in the activity and abundance of complexes I, II, III, and IV [152, 153] as well as the reduction of supercomplex-associated complex IV activity [153]. Cardiolipin is essential for activity of mitochondrial complexes and, in particular, complex I and complex IV. Defects of complex I are integral to the formation of ROS, whereas complex IV is essential for oxidative phosphorylation. In dogs with experimental HF, long-term therapy with elamipretide normalized mitochondrial respiration, mPTP opening, and membrane potential and improved the maximum rate of ATP synthesis [152] (Fig. 2). These improvements were accompanied by a reduction of cytochrome c release, a reduction of ROS formation, and improvements in mitochondrial complex I and IV activities [152]. Elamipretide also restored, albeit in part, protein levels of key subunits of mitochondrial complexes I through V [152]. Similar observations were made in LV tissue from humans with HF along with improvement of supercomplex-associated complex IV activity [153].

### Effects of elamipretide on LV systolic function

Studies in dogs with coronary microembolization-induced chronic HF with reduced LV ejection fraction (HFrEF) showed that 3 months’ monotherapy with daily subcutaneous injections of elamipretide improved LV systolic function and prevented progressive LV dilation without affecting heart rate, blood pressure, or systemic vascular resistance [152]. In these dogs, elamipretide significantly increased LV ejection fraction and LV fractional area of shortening compared with controls, and significantly reduced LV end-systolic volume and plasma concentration of n-terminal brain natriuretic peptide [152]. A phase 1/2 ascending single-dose study of elamipretide (4-h infusions, 0.005, 0.05, and 0.25 mg/kg/h) in 36 patients with HFrEF showed that elamipretide was well tolerated and significantly reduced LV end-diastolic (− 18 mL; *P* = 0.009) and end-systolic (− 14 mL; *P* = 0.005) volumes in the highest dose cohort and correlated with peak plasma concentrations, supporting a temporal association and dose–effect relationship [181]. No serious adverse events were reported in any of the cohorts and blood pressure and heart rate remained stable. In a more recent randomized phase 2 trial in patients with ischemic or idiopathic dilated cardiomyopathy receiving standard of care treatment for HFrEF, elamipretide was well tolerated but did not improve LV end-systolic volume compared with placebo [181]. None of the patients in this phase 2 trial had BTHS.

### Effects of elamipretide on Ca^2+^-ATPase and inflammatory cytokines

**Fig. 3:**
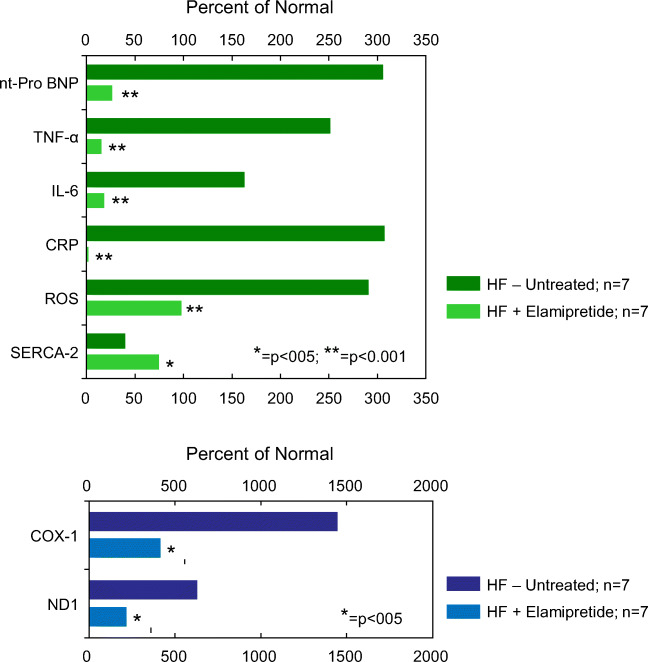
*Top*: Bar graph depicting magnitude of change of plasma cytokines, plasma natriuretic peptide, plasma reactive oxygen species (ROS), and left ventricular tissue levels of calcium ATPase (SERCA-2a) calculated as a percent of levels seen in normal dogs (Percent of Normal). The percentages are shown for untreated dogs with coronary microembolization-induced heart failure (HF-Untreated, *n* = 7) and for dogs with heart failure treated with elamipretide (HF+Elamipretide, *n* = 7). Original data in reference 151. nt-pro BNP, n-terminal pro-brain natriuretic peptide; TNF-α, tumor necrosis factor alpha; interlukin-6; CRP, c-reactive protein. *Bottom*: Bar graph depicting magnitude of change of two plasma mitochondrial fragments also referred to as damage-associated molecular patters (DAMPs) calculated as a percent of levels seen in normal dogs (Percent of Normal). CVOX1, subunit of cytochrome c oxidase (complex IV); ND1, subunit of complex I

In tafazzin knockdown mice, a model of Barth syndrome, Ca^2+^-ATPase or SERCA2a activity was impaired and SERCA2a tyrosine nitration increased compared with wild-type mice [87]. A reduction in SERCA2a expression often leads to poor LV active relaxation and overall LV diastolic dysfunction. Patients with myocardial noncompaction, typically present in Barth syndrome, almost always exhibit diastolic dysfunction [88]. These findings suggest that SERCA may be a viable therapeutic target for the cardiolipin deficiency typically observed in patients with Barth syndrome, particularly those manifesting HF with preserved ejection fraction (HFpEF). In a dog model of HFrEF, SERCA2a protein levels were shown to be significantly decreased in LV myocardium compared with normal dogs, but normalized after long-term treatment with elamipretide [152] (Fig. 3). The effects of elamipretide on diastolic LV function were also examined in a swine model of renovascular hypertension that manifests HFpEF, as evidenced by preserved LV ejection fraction, LV hypertrophy, poor LV relaxation and reduced Ca^2+^-ATPase activity, and expression and phospholamban phosphorylation at serine 16 [182]. In this swine model, treatment with elamipretide improved LV relaxation, ameliorated cardiac hypertrophy, and normalized phospholamban phosphorylation and Ca^2+^-ATPase activity and expression without affecting blood pressure or systolic LV function [182]. These results support the use of elamipretide as potential therapy for patients with Barth syndrome that manifest HFpEF.

Increased plasma levels of pro-inflammatory cytokines are a well-known feature of acquired HF and contribute to mitochondrial dysfunction and ultimately to the progressive worsening of the HF state. Given that BTHS is characterized by cardiomyopathies, it would not be surprising to observe increased levels of TNF-α and IL-6 in this disease state. Higher levels of TNF-α and IL-6 have been reported in BTHS and implicated in the pathophysiology of the disease. In dogs with microembolization-induced heart failure, plasma levels of the cytokines TNF-α, IL-6, and c-reactive protein (CRP) were significantly elevated compared with normal baseline levels [152]. In these HF dogs, levels of all 3 cytokines were normalized following long-term treatment with elamipretide [152] (Fig. 3). Elevated levels of plasma mitochondrial DNA DAMPs specifically within the COX1 and ND1 genes were markedly elevated in dogs with HF compared with normal baseline levels. In these HF dogs, long-term treatment with elamipretide normalized plasma levels of both COX1 and ND1 DAMPs (unpublished observation by the author) (Fig. 3).

### Elamipretide and skeletal muscle

As alluded to earlier, exercise intolerance is a hallmark of most, if not all, cardiomyopathies including those which occur in Barth syndrome. Patients with Barth syndrome have multiple signs of skeletal muscle impairments, including impaired functional exercise capacity and reduced daily activity, with a greater reliance on skeletal muscle glycolytic metabolism than those without Barth syndrome. This higher glycolytic capacity might be compensatory for an impaired capacity to generate ATP via oxidative phosphorylation. Mitochondrial respiration has been shown to be impaired in the skeletal muscle of Barth syndrome patients due to multiple factors that include mitochondrial supercomplex destabilization, higher degradation and reduced levels of mitochondrial cardiolipin, abnormal mitochondrial morphology, excessive production of reactive oxygen species, and defects in ATP synthase activity [50, 66, 131–133]. In dogs with HF, long-term treatment with elamipretide restored skeletal muscle fiber-type composition to a more normal distribution (increased proportion of skeletal muscle type 1 fibers relative to skeletal muscle type 2 fibers) [183]. Elamipretide also normalized skeletal muscle mitochondrial function as evidenced by significant greater improvements in mitochondrial respiration, membrane potential, and maximal rate of ATP synthesis when compared with untreated HF dogs [183]. In dogs with HF, therapy with elamipretide also increased skeletal muscle activity of cytochrome c oxidase (complex IV of the ETC) [182]. In a phase I/II multicenter, randomized, double-blind, placebo-controlled trial of elamipretide in 36 patients with genetically confirmed primary mitochondrial myopathy (not including Barth syndrome), participants who received elamipretide showed a significant dose-dependent increase in distance walked on the 6-min walk test (6MWT) compared with placebo [184]. These findings, when viewed in concert, suggest that therapy with elamipretide can improve skeletal muscle morphology and metabolism and, in doing so, potentially set the stage for an improvement of exercise tolerance in patients with Barth syndrome.

## Treating Barth syndrome with elamipretide

A phase 2/3 randomized, double-blind, placebo-controlled crossover trial followed by an open-label treatment extension of elamipretide in subjects with genetically confirmed BTHS (TAZPOWER Trial) is ongoing. The TAZPOWER Trial was designed to evaluate the efficacy and safety of the clinical-phase, investigational product elamipretide in patients with Barth syndrome; 12 patients (mean age 19.5 years, range 12 to 35 years) with genetically confirmed BTHS were enrolled. The phenotype in these patients was that of hypertrophic cardiomyopathy characterized by lower than normal LV end-diastolic volume and normal LV ejection fraction. In Part 1 of the trial, participants received once-daily subcutaneous injections of 40 mg elamipretide for 12 weeks, followed by a 12-week treatment with placebo, or vice versa, with a 4-week “washout” that occurred between each treatment. The second part of the ongoing trial involves an open-label extension for up to 168 weeks, in which 10 of the 12 patients participated. The primary objective, assessed at week 12, is the change from baseline in the 6MWT. Additional outcome measures include functional measures of muscle strength and heart performance, and subjective measures of patient-reported outcomes, as well as adverse effects.

Cardiac dysfunction has been shown to be a primary cause of early mortality in this patient population and improvements in left ventricular end-diastolic volume (LVEDV) and stroke volume are major determinants of peak exercise capacity in patients with hypertrophic cardiomyopathy. Recently reported trial results showed that elamipretide improves overall cardiac function, with averaged indexed cardiac stroke volumes increasing by 27%—from 40.8 mL at the start of the trial (baseline) to 51.8 mL—after 36 weeks of treatment with elamipretide in the open-label extension. In this study, elamipretide was generally well tolerated with the majority of adverse events being mild to moderate in severity. The most commonly reported adverse event was injection site reactions, occurring in 100% of patients while taking elamipretide [185, 186]. The U.S. Food and Drug Administration (FDA) has granted Fast Track and Orphan Drug designations for elamipretide for the treatment of patients with Barth syndrome. The encouraging clinical results seen in patients with BTHS treated with elamipretide support the potential use of this drug for the clinical management of this rare disease syndrome.
